# Gendered farmer perceptions towards soil nutrition and willingness to pay for a cafetière-style filter system for *in-situ* soil testing: Evidence from Central Kenya

**DOI:** 10.1016/j.heliyon.2024.e37568

**Published:** 2024-09-11

**Authors:** Philip Kamau, Ibrahim Ndirangu, Samantha Richardson, Nicole Pamme, Jesse Gitaka

**Affiliations:** a Directorate of Research and Development, Mount Kenya University, P.O. Box 342-01000, Thika, Kenya; b School of Natural Sciences, University of Hull, Cottingham Road, Hull, HU6 7RX, UK; c Department of Materials and Environmental Chemistry, Stockholm Univeristy, SE-106 91, Stockholm, Sweden

**Keywords:** soil nutrition, *in-situ* soil testing, microfluidic paper-based analytical devices, willingness to pay, cafetière-style filter system

## Abstract

Soil nutrition is a key pillar in agricultural productivity. However, point-of-need testing for soil nutrition is not readily available in resource-limited settings such as Kenya. We set out to study the perceived need for soil testing among farmers in this country. A group of 547 farmers from Murang'a and Kiambu counties in central Kenya were recruited through multi-stage sampling to help assess the perceptions and willingness to pay (WTP) toward a prototype technology for surveillance of *in-situ* soil nutrition. The technology is based on a cafetière-style filter system for extraction and a microfluidic paper-based analytical device (μPAD) for nutrient readout. We employed the double bounded choice contingent valuation method (CVM) to analyze the willingness of farmers to accept and pay for the prototype if the technology was available on the market. It was found that currently, only 1.5 % of farmers carry out soil testing. The high costs of analysis at testing centers, which are often far from the farmers, are among the main reasons contributing to the majority of farmers not testing their soils. The farmers surveyed were generally willing to make their soil data publicly accessible, especially to extension officers. CVM showed that uncontrolled WTP had a 94.24 % premium above KSh1,000 ($6.60) incurred by using the existing rapid testing method. Factoring the control variables and disaggregating the model into gender categories, the findings showed that youth, women, and men had WTP values of KSh1,612.53 ($10.75), KSh1,558.68 ($10.39), and KSh1,504.83 ($10.03), respectively, indicating that farmers can indeed pay for the convenience to test their soils *in situ*. Through the democratization of soil nutrition data, extension agents can enhance the improvement of agricultural productivity, which implies that farmers can commercialize their agricultural activities.

## Highlights

• Low occurrence of soil testing in Kenya due to lack of knowledge, distance to laboratory, and costs.
• A cafetière-style filter system with a colorimetric μPAD readout was investigated as a solution to this challenge.
• Young farmers, in particular, demonstrate a high willingness to pay for the cafetière-style filter system technology.

## 1 Introduction

Poor soil nutrition is a major factor that negatively influences agricultural productivity in sub-Saharan Africa (SSA) [[1], [2], [3], [4], [5], [6]]. Soil malnutrition affects over 350 million hectares in SSA, with soils mostly deficient in nitrogen (N) and phosphorus (P) [2,7]. Legumes yield below 1 ton per hectare (t ha^−1^) despite their capacity to produce over 2 t ha^−1^. Similarly, cereals yield approximately 1.5 t ha^−1^ when they can make above 5 t ha^−1^ [2]. With the dwindling farm sizes and the impact of climate change in SSA, we must look for possible agricultural intensification strategies to ensure high production that serves the urban and rural populations as well as export markets [[8], [9], [10]]. One of the enabling techniques is to enhance soil fertility in order to improve agricultural productivity. However, relatively few farmers are currently conducting soil testing. This is because the process is expensive and also because the testing laboratories are often located at a considerable distance from the farms [11].

Trained personnel generally conduct laboratory soil test procedures [12]. They use reagents to extract available ions from the soil. UV/vis absorption spectroscopy or inductively coupled plasma atomic emission spectroscopy (ICP-AES) are common detection methods to measure ion concentrations [13]. These laboratory soil testing procedures are carried out by trained personnel, and the workflow takes some time. Consequently, farmers need to wait a few days to weeks before they get their results [14].

Many farmers in Sub-Saharan Africa (SSA) lack soil testing knowledge, dependable testing services, or adequate laboratories [11]. National agricultural research organizations mainly offer soil testing services. Other service providers include private companies, such as Crop Nutrition Laboratory Services Ltd. (CROPNUTS), and universities, such as Makerere University (Uganda) and the University of Nairobi (Kenya) [15,16]. The high cost of soil testing and the extremely sparse distribution of laboratories discourage peasant farmers from accessing soil testing services [15]. Furthermore, current information on soil nutrients provides an erratic representation of heterogeneous and dynamic environments because soil testing facilities are relatively inaccessible to smallholder farmers [17].

The sparse laboratory services in SSA have prompted the development of *in-situ* rapid methods that enable soil testing [18]. This includes non-liquid spectroscopy-based nutrient testing systems that have gained acceptance in SSA [15]. The AgroCares nutrient scanner (AgroCares, NL) has seen expanded usage in Eastern Africa, particularly in Tanzania, Rwanda, Burundi, Kenya, and Uganda on account of its soil testing rapidity. Farmers are given soil nutrient results and soil improvement recommendations within a few hours [19]. Whilst spectroscopy-based scanners are expensive, they have a long work life. Since the scanners use buttons, it is relatively easy to train farmers to operate them compared to colorimetric methods with reagents. Despite all the advantages of these AgroCares scanners in helping farmers test their soil on-site, their initial cost of several thousand US dollars is above affordability by many SSA farmers.

The rising literature on willingness to pay (WTP) and uptake of new agricultural technology often does not take into consideration factors that influence gender-specific needs and preferences among men, women, and younger farmers [[20], [21], [22], [23], [24], [25], [26], [27], [28]]. Also, most of the studies that address WTP do not consider gender-specific factors that might affect the willingness and perceived needs for the new technologies [[20], [21], [22],^24^,^25^,[28], [29], [30]]. Farmers who are women and young adults (18–35 years) are faced with greater challenges in affording and adopting new technologies than their male counterparts [[31], [32], [33]]. This is because women and younger farmers typically have low access to agricultural training and education, low assets such as livestock, farm machinery, and implements, and biased access to credits [34,35]. Lack of attention to gender-specific needs causes gender inequalities in technology adoption which may enlighten stakeholders about the low uptake of new technologies across the agricultural sector [36,37]. This is worsened by a general exclusion of women and youthful farmers from decision-making on matters pertinent to agriculture [31,38].

**Fig. 1:**
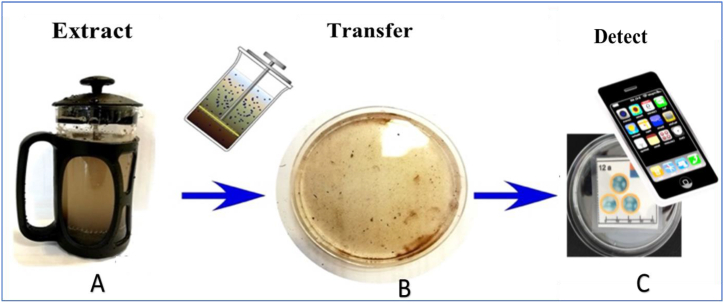
Proposed prototype, which uses a cafetière for soil filtration and water-soluble nutrient extraction (available nutrients) combined with a microfluidic paper-based analytical device (μPAD). **(A)** Soil and water are added to the cafetière and mixed using the plunger. **(B)** The soil is then trapped below the cafetière's filter mesh, and the liquid is decanted for further analysis by a μPAD. **(C)** The color change on the μPAD is recorded using a smartphone app for NPK and pH analysis results. (For interpretation of the references to color in this figure legend, the reader is referred to the Web version of this article.)

To address this, we investigated farmers' and key stakeholders’ current practices and perceived needs concerning soil analysis and health. We targeted men, women, and young farmers to capture data from these groups. Linked to this, we explored the WTP for a low-cost technology (Fig. 1) that would enable a farmer to monitor soil nutrition on their own land.

The proposed low-cost workflow is based on a cafetière-style nutrient extraction system with a paper microfluidic sensor for colorimetric readout (Fig. 1) [26,28]. Fig. 1(A) shows a cafetière with a plunger for mixing soil and water to extract macronutrients. Fig. 1(B) depicts a shallow dish that collects the liquid decanted from the cafetière. A mobile phone application can then be employed to photograph the μPAD for colour-based nutrient analysis (Fig. 1(C)). This simple-to-use system would enable simple *in-situ* soil testing carried out by individual farmers, who could then submit their results to central databases to support a wider understanding of soil health country-wide. The system is designed to follow the World Health Organization (WHO) ASSURED criteria to enable everyone in limited-resource countries access to diagnostics and analysis [39,40]. This approach ensures measurement systems are **a**ffordable, **s**ensitive, **s**pecific, **u**ser-friendly, **r**apid, **e**quipment-free, and **d**elivered to those who need it. These criteria often apply to microfluidic paper-based analytical devices (μPADs) [[20], [21], [22]]. The μPADs are simple to fabricate, run power-free, are low cost, sensitive, and easy to dispose of [41,42]. The μPADs method thus has prospects of meeting the point of need for SSA farmers.

Our μPAD is modified with chemical reagents to provide a colorimetric readout of water-soluble nutrients, which can be captured using a mobile phone. Such a system would have a low set-up cost, with up to KSh1,000 ($6.60) for a cafetière that is procured once and an estimated KSh100 ($0.70) to procure a set of 5 μPADs from registered distributors any time a farmer wants to test their soil nutrients. The recurring cost of procuring μPADs is relatively cheaper than accessing the existing rapid soil testing technologies, which cost at least KSh1,000 ($6.60), and the conventional laboratory testing procedure, which costs at least KSh1,500 ($10). The initial cost of a cafetière purchase can be mediated by procurement through farmer groups via a cost-sharing approach. The advantage of the proposed system over conventional soil testing practices is that farmers can test the soil nutrition on different zones of their farmland based on zonal productivity history.

This study aimed to further our understanding of farmers' knowledge of soil nutrition, the need for new low-cost technology, and the willingness to democratize data collected, which is typically not addressed in other studies. We aimed to investigate whether farmers would be willing to share their soil nutrition data and whether this willingness varied between gender or age. As such, in case of successful development of an *in-situ* soil testing technology, farmers' trusted persons will be instrumental in targeted technology dissemination. The study also scoped perceptions and motivations of stakeholders, *i.e.*, farmers and government officers, in the democratization of soil data in Kenya. We aimed to answer the following research questions: (1) What are local farmers' and key stakeholders’ current practices and perceived needs concerning soil analysis and health? (2). What are the gendered factors contributing to the low uptake of soil testing in Kenya? (3) Will farmers across genders be willing to accept and pay for a power-free cafetière-style extraction and analysis system? (4) Will farmers across genders democratize the farm soil nutrition data?

## 2 Materials and methods

### 2.1 Study area

**Fig. 2:**
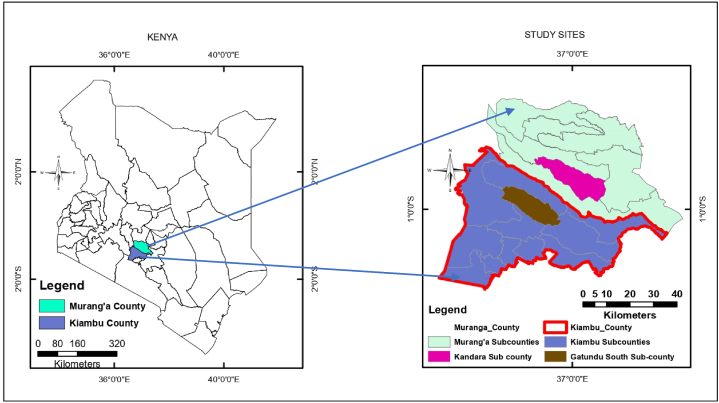
Map of the study area in the sub-counties of Gatundu south and Kandara within Kiambu and Murang'a counties of Kenya. The map was generated using ArcGIS 10.5.

The survey was conducted in Gatundu south and Kandara sub-counties of Kiambu and Murang'a counties of Kenya, respectively (Fig. 2). Agricultural activities in both study sites fall under different agroecological zones (AEZs) ranging from AEZ I (Agro-Alpine) to AEZ III (Medium Potential) [10,43].

Gatundu South sub-county has a population of 121,693 persons from 31,472 households and covers 192.4 km^2^ [44]. The sub-county receives a bimodal rainfall pattern, with the first peak of long rains occurring between March to May, while the second peak of short rains occurs between October and December. The precipitation in the area exceeds 2000 mm, with annual temperatures averaging 18–22 °C. A mixture of deep and well-drained reddish-brown Rhodic Nitisols and Humic Nitisols soils are found in the area [45]. These soils support the cultivation of multiple crops, including food crops (e.g., potatoes, beans, and maize), tropical fruits (e.g., avocado, oranges, and pawpaw), coffee, and tea. Also, farmers engage in livestock production as a diversified livelihood strategy.

Kandara sub-county has a population of 175,098 persons from 50,704 households and covers an area of 193.6 km^2^ [44]. The sub-county receives rainfall in a bimodal pattern with precipitation of above 2600 mm. The long rains in this area start towards the end of March, hit the highest precipitation in April, and begin to reduce towards the end of May. The short rains commence in October, with the highest precipitation experienced in November. The annual temperatures in the Kandara sub-county are 18–21 °C. The area has Humic Nitisols soils that are characterized by acidic topsoil, dark reddish-brown color, extremely deep, and a well-drained profile [46]. Farmers engage in mixed crop-livestock production systems.

### 2.2 Sampling design and sample size

Ethical approval for the study was granted through the Mount Kenya University Ethical Committee, ref MKU/ ERC /1797. A multistage sampling procedure was employed to establish the study area and sample size. The choice of these counties was informed through discussion with local agricultural officers, taking into account the proximity to the largest food market (Nairobi) and high land fragmentation due to population growth and soil fertility loss from suboptimal use of fertilizers in a set-up of continuous cropping and climate change [[8], [9], [10]]. A similar approach was used to select the Ndarugu and Ruchu wards of Gatundu South and Kandara sub-counties, respectively. Extension officers of Ndarugu and Ruchu agricultural wards supplied a list of 4500 and 4000 farmers from all villages, from households headed by men and women, respectively. In our study, households became the basic element of the survey and were randomly selected.

The sample size of the study was acquired using the Cochran formula as explained by Israel [47]. The formula is presented in Equation (1);

(1) $$n=\frac{Z^{2}\left(pq\right)}{e^{2}}\approx \frac{0.96^{2}*0.5*0.5}{0.425^{2}}\approx 532\;\text{households}$$

where n = sample size, Z = standard error associated with the chosen level of confidence, p = estimated proportion of an attribute present in the population (variability), q = 1−p, and e = acceptable sample error. Since there is no credible documented variability of farmers in both counties, the level of precision p is assumed to be maximum (0.5). The values of Z (at 95 % confidence level) and *e* used were 1.96 and 0.425, respectively. The sample size was approximately 532 households, and probability proportion to size criteria allotted 282 and 250 households to Ndarugu and Ruchu, respectively. We scaled up the sample size to 547 (Table 1) to absorb the risk of possible spoiled questionnaires that could arise from misinformation or non-response. In the Ndarugu ward, the youth category comprised 46 men-headed and 10 women-headed households. In contrast, in the Ruchu ward, the composition was 31 men-headed and 7 women-headed households within the youth category.

**Table 1:** Sampled men and women-headed households sampled per sub-county.

Ward	Men (35+)	Women (35+)	Women youth (18–35)^a^	Men youth (18–35)^a^	Total
Ndarugu	172	62	10	46	290
Ruchu	170	49	7	31	257
Total	342	111	17	77	547

a Values in parenthesis are years of age.

### 2.3 Data collection and processing

The cross-section survey used a semi-structured questionnaire (**ESI 1**) to collect household data, including institutional factors, socioeconomic factors, demographics, existing farming practices, perceptions towards a portable *in-situ* soil surveillance technology system, and soil testing knowledge. The formulated questionnaire was programmed in Open Data Kit (ODK) software for electronic data collection [30,31]. Before the data collection exercise began, the training of enumerators was done, and the questionnaire was pretested using 41 households in the Ithiru ward of Kandara sub-county on June 17, 2021, in a similar AEZ as the study sites. All enumerators were trained before the administration of the questionnaires. It was ensured that the principles of anonymity and voluntary participation were upheld. The data collectors received training to obtain informed consent from the farmers and confirm their willingness to participate in the study. The main data collection exercise took place between June 19, 2021 and July 4, 2021.

Cleaning of data was carried out with SPSS v.23 which was also used for the actual analysis along with STATA v.15. The analysis was performed through disaggregation of the findings into gender. For continuous variables, t-tests were conducted to identify significant differences, and Chi-square tests were employed to determine the independence among categorical variables. The econometric model double bounded dichotomous choice–contingent valuation method (CVM) was analyzed using STATA.

### 2.4 Theoretical foundation and analytical framework

#### 2.4.1 Theoretical foundation

An acceptable scientific method of evaluating the non-market products’ (good and service) value is via the use of monetary terms technique. The valuation gives a reflection of the perceived impact that the products might have on the welfare of consumers contingent on the products being in the market. Theoretically, the economic value of a product can be measured in four ways, holding utility constant, as proposed by Hicks [48]. According to the Hicksian theory, the welfare measurement entails the assessment of compensating variation and compensating surplus; a method that measures losses or gains compared to the primary utility level of a market product. The theory also measures equivalent surplus and equivalent variation to assess losses and gains attached to a prospective alternative level of utility. Measures of variation are only used for changes in product price such that individuals respond by varying the consumption of products of interest [49]. Measures of surplus apply when the changing factor is the product quality or quantity but consumers can just purchase fixed quantities [50]. Freeman [50] alludes that most applications of Hicksian theory entail fixed variations (increases and decreases) in the quality and quantity of non-market products. In our case, we adopt the measurements of human welfare via Hicksian welfare surplus, specifically the compensating surplus, as derived in Equation (2).

(2) $$u\left(Q^{0},M^{0}\right)=u\left(Q^{1},M^{0}-CS\right)$$

where u is the indirect utility function, Q is the non-market product, M is income or money, and CS is the compensating surplus. This means that farmers will be willing to accept/pay for an *in-situ* soil nutrition surveillance technology as an indicator of the acquisition of positive change.

#### 2.4.2 Econometric modelling for assessing willingness to pay for portable analysis system

The willingness to pay for an *in-situ* soil nutrition surveillance tool was measured using an econometric model known as the double bounded contingent valuation method (CVM) [29,51,52]. Research in this sense evaluates products or services not yet on the market, so farmers were asked to value them based on there being a market [53]. This analysis aimed to determine if farmers would be willing to pay for the convenience value of a rapid soil diagnostics system that would enable *in-situ* testing compared to the current price of approximately KSh1,000 ($6.67) using the existing testing methods. The CVM model developed for this study is elaborated in the supplementary section **ESI 2** and variable specifications are in **ESI 3**. The contingent valuation method (CVM) model was tested for multicollinearity to identify if the explanatory variables were inter-correlated. We used the Variance Inflation Factor (VIF) such that ${VIF}_{i}=1/1-R_{i}^{2}$, where $R_{i}^{2}$ represents an *R*^*2*^ of an artificial Ordinary Least Square and assumes that each explanatory variable is dependent on others. The individual and mean VIF values were below 10 as presented in **ESI 4** implying that multi-collinearity was not an econometric problem with the model data.

## 3 Results

Targeted soil testing interventions require evidence data from smallholder farmers. As such, the findings of this research entailed farmer and farm characteristics, farmers’ knowledge about soil nutrition, existing soil nutrition management practices, the need for soil analysis, and the willingness to pay for the cafetière-style soil testing technology.

### 3.1 Gendered farmer and farm characteristics

The findings are summarized in Table 2, Table 3. Out of the 547 farmers surveyed, the largest group (46.4 %) had only attained up to primary education followed by 34.6 % who had secondary education. Most (82.1 %) of the farmers received a low monthly income (KSh1 – 15,000), including 92.8 % of the women sampled. Forty-two percent of the household heads were above the age of 55 years. More young adults and men earned off-farm income compared to the women, and the difference was statistically significant (p < 0.01). Most (92.3 %) of the farmers own title deeds for land ownership. More men than women and young adults used irrigation in crop production. Regarding asset ownership, the young adults had a statistically lower value of assets than the men and women gender categories. Women significantly received more extension contacts than men and young adults. Distance to the market did not significantly differ among the gender groups. Men (>35 years) cultivated larger crop areas than women and young adults. Table 3 shows that ownership of livestock was not different among the gender groups. Also, the average household size was about four persons per household and significantly differed among the study arms (p < 0.01).

**Table 2:** Gendered descriptive statistics for farmer and farm characteristics (categorical variables).

Variable	Pooled Freq (%)	Men Freq (%)	Women Freq (%)	Youth Freq (%)	χ^2^
*Education*					
No formal education	31 (5.67)	14 (4.09)	16 (14.41)	1 (1.06	61.552∗∗∗
Primary	254 (46.44)	166 (48.54)	65 (58.56)	23 (24.47)	
Secondary	189 (34.55)	115 (33.63)	26 (23.42)	48 (51.06)	
College	61 (11.15)	40 (11.70)	4 (3.60)	17 (18.09)	
University	12 (2.19)	7 (2.05)	0.00	5 (5.32)	
*Employment*					
Student	11 (2.01)	3 (0.88)	3 (2.70)	5 (5.32)	15.234∗∗
Self-employed	518 (94.70)	329 (96.20)	107 (96.40)	82 (87.23)	
Formal employment	18 (3.29)	10 (2.92)	1 (0.90)	7 (7.45)	
*Income*					
None (student)	2 (0.37)	0.00	0.00	2 (2.13)	27.106∗∗∗
None (Non student)	2 (0.37)	2 (0.58)	0.00		
Low (Kes1-15,000)	449 (82.08)	265 (77.49)	103 (92.79)	81 (86.17)	
Middle (KSh15,000–50,000)	84 (15.36)	66 (19.30)	7 (6.31)	11 (11.70)	
High (>KSh50,000)	10 (1.83)	9 (2.63)	1 (0.90)		
*Age*					
18–35 years	94 (17.18)	0.00	0.00	94 (100)	547.765∗∗∗
36–55 years	223 (40.77)	172 (50.29)	51 (45.95)	0.00	
>55 years	230 (42.05)	170 (49.71)	60 (54.05)	0.00	
Off-farm income (Yes = 1)	157 (28.70)	111 (32.46)	9 (8.11)	37 (39.36)	30.579∗∗∗
Group membership (Yes = 1)	406 (74.22)	250 (73.10)	88 (79.28)	68 (72.34)	1.883
Credit (Yes = 1)	227 (41.50)	148 (43.27)	58 (52.25)	21 (22.34)	19.943∗∗∗
*Land tenure*					
Leased	6 (1.10)	3 (0.88)	1 (0.90)	2 (2.13)	28.449∗∗∗
Own title	505 (92.32)	321 (93.86)	109 (98.20)	75 (79.79)	
Both	36 (6.58)	18 (5.26)	1 (0.90)	17 (18.09)	
Irrigation	223 (40.77)	143 (41.81)	35 (31.53)	45 (47.87)	6.041∗∗
					

Values in parenthesis are percentages; ∗∗∗ and ∗∗ are statistically significant at 1 % and 5 %, respectively. The significances of differences were computed using Pearson's χ^2^ test from a crosstabulation analysis.

**Table 3:** Gendered descriptive statistics for farmer and farm characteristics (continuous variables).

Variable	Pooled Mean (SD)	Men Mean (SD)	Women Mean (SD)	Youth Mean (SD)	F-value
Assets (Ksh)	40,4220.5 (31,0475.4)	43,9530.50 (33,8555.70)	36,0833.30 (28,2293.30)	32,7600 (19,7149.40)	1.030
Extension education (No. of times)	0.4 (0.7)	0.45 (0.72)	0.36(0.69)	0.27 (0.58)	2.470∗
Market distance (km)	2.41 (3.66)	2.39 (3.51)	2.5 (3.97)	2.37 (3.86)	0.430
Crop area (acres)	1.12 (0.98)	1.2 (1.06)	1.12 (0.95)	0.81 (0.61)	5.607∗∗∗
TLU^a^	0.77 (0.51)	0.81 (0.53)	0.75 (0.47)	0.69 (0.46)	2.159
HH size (number of people)	3.82 (1.66)	4.1 (1.41)	3.96 (1.68)	3.15 (1.66)	11.808∗∗∗
N	547	342	111	94	

TLU represents Tropical Livestock Units; HH represents household; SD denotes standard deviation; Values in parenthesis are standard deviations; N represents the sample size; ∗∗∗ and ∗∗ mean that the differences computed from One-way ANOVA are statistically significant at 1 % and 5 %, respectively.

a denotes the values used in computing TLU (**ESI 3,**Table ESI1).

### 3.2 Existing knowledge about soil nutrition across genders

We studied the farmers’ knowledge of soil dynamics (e.g. pH, soil losses, and nutrients), and perceived nutrient levels in their farms (Table 4). The survey revealed that more young farmers (72.3 %) had prior knowledge of soil pH compared to older men (57 %) and women (55.9 %). The majority (66.7 %) of the farmers considered their soils fairly fertile.

**Table 4:** Gendered descriptive statistics for soil nutrition knowledge.

Variable	Pooled	Men	Women	Youth	χ^2^
	Freq (%)	Freq (%)	Freq (%)	Freq (%)	
*Knowledge*					
of soil nutrients	252 (46.07)	153 (44.74)	47 (42.34)	52 (55.32)	4.102
of soil pH	325 (59.52)	195 (57.18)	62 (55.86)	68 (72.34)	7.803∗∗
of soil loss	510 (92.24)	322 (94.15)	101 (90.99)	87 (92.55)	1.412
*Perceived nutrient levels on own farm*					
Very poor	5 (0.91)	4 (1.17)	0.00	1 (1.06)	9.223
Poor	58 (10.60)	45 (13.16)	7 (6.31)	6 (6.38)	
Fair	365 (66.73)	218 (63.74)	78 (70.27)	69 (73.40)	
Good	113 (20.66)	72 (21.05)	24 (21.62)	17 (18.09)	
Very good	6 (1.10)	3 (0.88)	2 (1.80)	1 (1.06)	
N	547	342	111	94	

Values in parenthesis are percentages; N represents the sample size; ∗∗ indicates a significant difference at a 5 % level computed using Pearson's χ^2^ test from a crosstabulation analysis.

### 3.3 Existing practices to mitigate soil fertility losses across genders

We investigated the existing practices to mitigate soil fertility losses (Table 5). A slight majority (58 %) used inorganic fertilizers. Diammonium phosphate (DAP) and nitrogen, phosphate, potassium (NPK) (17:17:0) were the two most used basal fertilizers at 20.8 % and 16.1 %, respectively. Calcium ammonium nitrate (CAN) was the most used top-dressing fertilizer. Other farmers used farmyard manure (FYM) (37.9 %), compost manure (1.5 %), and industrial organic fertilizers (0.2 %) among other minor methods (0.8 %) such as residues, crop rotation, and crop cover. Despite the majority (59.4 %) of farmers knowing that their soils could be acidic, only approximately 30 % took measures to control condition. A proportion of 17.2 % of the farmers who controlled low pH levels mixed FYM with ash, while another 8.8 % used lime, especially on their coffee and tea farms.

**Table 5:** Gendered descriptive statistics for the existing fertility management practices.

Variable	Pooled	Men	Women	Youth	χ^2^
	Freq (%)	Freq (%)	Freq (%)	Freq (%)	
*Mitigation of soil fertility loss*					
Inorganic fertilizers	317 (57.95)	197 (57.60)	76 (68.47)	44 (46.81)	28.882∗∗
*Basal fertilizers*					
DAP	114 (20.84)	69 (20.18)	26 (23.42)	19 (20.21)	9.335∗∗∗
NPK (17:17:0)	88 (16.09)	62 (18.13)	18 (16.22)	8 (8.51)	
NPK (23:23:0)	20 (3.66)	12 (3.51)	6 (5.41)	2 (2.13)	
NPK (25:5:5)	17 (3.11)	11 (3.22)	4 (3.60)	2 (2.13)	
NPK (25:25:25)	15 (2.74)	6 (1.75)	7 (6.31)	2 (2.13)	
NPK (20:20:0)	7 (1.28)	3 (0.88)	2 (1.80)	2 (2.13)	
Mavuno basal	6 (1.10)	4 (1.17)	2 (1.80)	0.00	
Others	4 (0.73)	4 (1.17)	0.00	0.00	
*Topdressing fertilizers*					
CAN	42 (7.68)	25 (7.31)	11 (9.91)	6 (6.38)	0.779
UREA	4 (0.73)	1 (0.29)	0.00	3 (3.19)	
Manure	217 (39.67)	135 (39.47)	33 (29.73)	49 (52.13)	
Compost	8 (1.46)	8 (2.34)	0.00	0.00	
Crop cover, residues, rotation	4 (0.73)	1 (0.29)	2 (1.80)	1 (1.06)	
Industrial organic fertilizer	1 (0.18)	1 (0.29)	0.00	0.00	
*Control of soil pH*					
No control	379 (69.29)	242 (70.76)	76 (68.47)	61 (64.89)	32.841∗∗
Wood ash	94 (17.18)	42 (12.28)	28 (25.23)	24 (25.53)	
Liming	48 (8.78)	38 (11.11)	5 (4.50)	5 (5.32)	
Organic manure	10 (1.83)	9 (2.63)	0.00	1 (1.06)	
Fallowing	7 (1.28)	5 (1.46)	1 (0.90)	1 (1. 06)	
Mulching	5 (0.91)	3 (0.88)	1 (0.90)	1 (1. 06)	
Crop rotation	4 (0.73)	3 (0.88)	0.00	1 (1. 06)	
N	547	342	111	94	

Values in parenthesis are percentages; N represents the sample size; ∗∗∗ and ∗∗ are statistically significant at 1 % and 5 %, respectively, computed using Pearson's χ^2^ test from a crosstabulation analysis.

### 3.4 Soil analysis needs

A minority (1.5 %) of the farmers currently have their soil tested for nutrients and pH. Only 4.4 % of the sampled farmers were aware of existing rapid soil diagnostic technology whereby they were referring to the AgroCares Nutrient Scanners (Table 6).

**Table 6:** Current soil testing capacity and awareness of rapid testing technologies.

Variable	Pooled	Men	Women	Youth	χ^2^
	Freq (%)	Freq (%)	Freq (%)	Freq (%)	
Soil testing	8 (1.46)	8 (2.34)	0.00	0.00	4.866^a^
Aware of any existing rapid soil test method	24 (4.39)	18 (5.26)	4 (3.60)	2 (2.13)	1.932
N	547	342	111	94	

Values in parenthesis are percentages; N represents the sample size.

a denotes statistically significant at 10 %, computed using Pearson's χ^2^ test from a crosstabulation analysis.

**Fig. 3:**
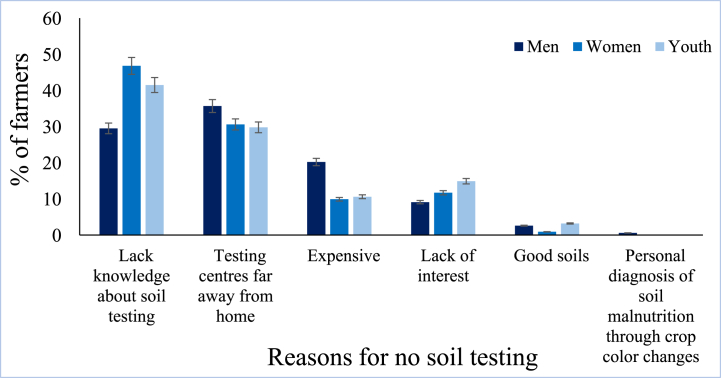
Reasons given by the surveyed farmers for not testing their soils (n = 547). The error bars show differences in the percentage of farmers across three groupings based on the reasons provided. To explain these visualized differences, a chi-square test was done (**ESI 6**). In general, the percentage of farmers who indicated various reasons for not testing their soil had significant differences across the three groupings (χ^2^ = 22.280, p = 0.014).

Farmers cited different reasons for not testing their soils, for instance, lack of knowledge about soil testing, including not knowing who tests the soil, what is tested in the soils, why soils should be tested, how to sample soils, and where to take a soil sample for testing. More men (35.7 %) than women (30.6 %) and young adults (29.8 %) cited that the testing centers were far away from the households. Other farmers quoted reasons such as soil testing being an expensive process (16.5 %), lack of interest in soil testing (10.6 %), and that the soils were already good and needed no testing (2.4 %). Fig. 3 represents the reasons that farmers gave for not testing their soils.

### 3.5 Perceptions about a portable, rapid soil testing, and power-free cafetière-style system

We gave farmers a description and the workability of the potential affordable rapid testing technology for *in-situ* soil nutrition via a cafetière-style filter system. All the farmers across gender groups thought they had the capability to use it (Table 7). In addition, we explained to farmers that the potential rapid testing solution could cost approximately $6–10, and they perceived the technology as affordable. The majority of farmers did not find any barrier that could hinder their use of the proposed technology. A minority (3.8 %) cited that the initial cost of the prototype and its complexity (1.8 %) would be a barrier to its use. A vast majority (96.7 %) expressed their interest in trialing the in-situ soil nutrition surveillance technology, while a slightly lower percentage (96.3 %) of farmers expressed their willingness to pay for it. Some of the few farmers who expressed their unwillingness to pay for rapid soil testing technology cited that their purchasing drive would be dependent upon a successful usage of the technology by other farmers.

**Table 7:** Perceptions about *in-situ* soil nutrition surveillance technology.

Variable	Pooled	Men	Women	Youth	χ^2^
	Freq (%)	Freq (%)	Freq (%)	Freq (%)	
*Perceptions about the proposed soil nutrition surveillance technology*					
If a farmer thinks technology is affordable	536 (98)	339 (99.1)	104 (93.7)	93 (98.9)	13.052∗∗∗
*Barriers to the use of the technology*					
None	489 (89.4)	316 (92.4)	88 (79.3)	85 (90.4)	30.534∗∗∗
Initial cost	21 (3.8)	13 (3.8)	3 (2.7)	5 (5.3)	
Other	16 (2.9)	7 (2)	7 (6.3)	2 (2.1)	
Sounds complex	10 (1.8)	3 (0.9)	7 (6.3)	1 (1.1)	
No formal education	11 (2)	3 (0.9)	6 (5.4)	1 (1.1)	
*Interest and willingness*					
Interested in trialing technology	534 (96.7)	337 (98.5)	105 (94.6)	92 (97.9)	5.647∗
Willing to purchase technology	527 (96.3)	334 (97.7)	102 (91.39)	91 (96.8)	7.987∗∗

Values in parenthesis are percentages; N represents the sample size; ∗∗∗, ∗∗, and ∗ are statistical significance at 1 %, 5 %, and 10 %, respectively, computed using Pearson's χ^2^ test from a crosstabulation analysis.

We further used CVM to quantify farmers' willingness to pay for the proposed portable rapid soil testing method after the majority cited that the technique can be affordable and were willing to purchase it. The findings of the CVM model with no control variables are presented in Table 8. The model positions farmers' willingness to pay (WTP) at KSh1,942.37 ($12.95). This indicates that most sampled farmers accepted the initial and upper bids that bore 10 % and 20 % premiums, respectively. The perceived need for soil testing placed farmers’ WTP at a 94.24 % premium above the KSh1,000 ($6.67) they incur using the existing rapid testing method.

**Table 8:** Estimated contingent valuation method without control variables.

Variable	Coef.	Std. Err.	z	P>|z|	[95 % Conf. Interval]	
β_cons	1942.37^a^	166.78	11.65	0.000	1615.49	2269.26
σ_cons	544.26^a^	113.72	4.79	0.000	321.37	767.15
Log-likelihood	−213.389					

a Is significant at 1 % (N = 547). The coefficients were generated using the *doubleb* Stata module that uses maximum likelihood estimation (MLE) to get estimates for β and σ.

**Fig. 4:**
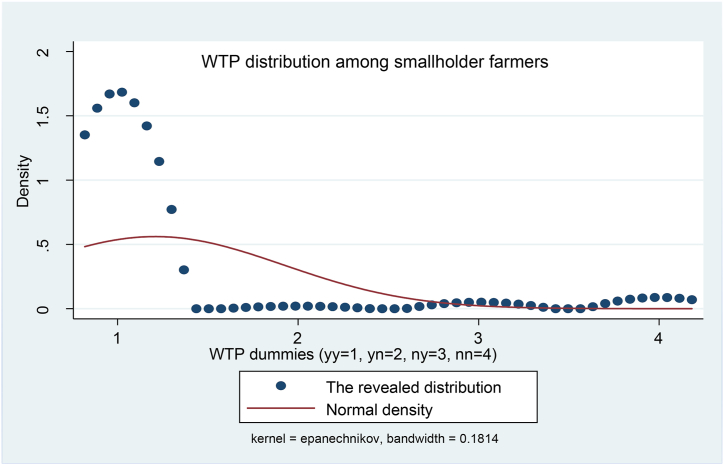
Distribution of willingness to pay for *in-situ* soil nutrition surveillance technology. The nonparametric kernel density estimation was performed using the *kdensity* command in Stata. The distribution is highly skewed to the right indicating a great need for an alternative soil testing method. yy = 1 means that the first and second responses to the first and the second bids were both “yes”; yn = 2 means that the first and second responses to the first and the second bids were “yes” and “no” respectively; and ny = 3 means that the first and second responses to the first and the second bids were “no” and “yes” respectively; and nn = 4 means that the first and second responses to the first and the second bids were both “no” (see **ESI 2**).

The distribution of WTP is shown in Fig. 4, which depicts the need for soil testing among farmers. The concentration of distribution density is around 1 (one), implying that the farmers accepted the first (10 % premium) and the second (20 % premium) bids. This implies that willingness to pay goes beyond the 20 % premium over the cost ($6.67) of the existing rapid test.

The overall WTP obtained from a controlled CVM model (Table 9) was KSh1,534.28 ($10.23), which is KSh400 ($2.37) less than the uncontrolled WTP (see Table 8). After gender disaggregation, the young adult farmers had the highest WTP value of KSh1,612.53 ($10.75) for the new technology. Men had the lowest value of WTP at KSh1,504.83 ($10.03).

**Table 9:** Gendered estimated WTP with control variables.

Gender	Variable	Coef.	Std. Err.	z	P>|z|	[95 % Conf. Interval]	
Pooled	WTP	1534.28^a^	210.94	7.27	0.000	1120.84	1947.72
Men	WTP	1504.83^a^	250.10	6.02	0.000	1014.65	1995.02
Women	WTP	1558.68^a^	248.06	6.28	0.000	1072.50	2044.86
Youth	WTP	1612.53^a^	272.53	5.92	0.000	1078.38	2146.68

a Is statistical significance at 1 %; WTP stands for willingness to pay (N = 547). The coefficients were generated using the *doubleb* module in Stata that uses maximum likelihood estimation (MLE) to get estimates for β and σ.

#### 3.5.1 Influence of control variables on WTP for the new soil testing technology using contingent valuation method

Primary education, post-primary education, self-employment, high income (>KSh50,000 ($333.33)), household size, age (36–55 years), age (above 55 years), off-farm income, distance to market, and tropical livestock unit (TLU) were the significant determinants of willingness to pay for the proposed soil testing cafetière-style filter system. The findings are presented in **ESI 5**. Gender was a significant determinant of WTP (p < 0.05), in which men positively influenced WTP as expected in **ESI3**. Literacy proficiency influenced WTP positively (p < 0.01), implying that WTP increases with education levels. Self-employment on the farm (agribusiness) had a significant positive influence on WTP (p < 0.05). The higher the farmer's income from all sources, the lower their WTP for agricultural technologies (p < 0.01). A larger household size reduced farmers' WTP for the proposed simple analytical tool for *in-situ* soil surveillance (p < 0.01). Willingness to pay for an *in-situ* soil nutrition surveillance technology was influenced positively by age, but the influence was reduced as age advanced (p < 0.01). Farmers who received off-farm income have lower WTP for the simple *in-situ* soil nutrition surveillance technology than their counterparts who did not receive off-farm income (p < 0.1). Longer distances to the nearest input/product market significantly lower WTP for an *in-situ* soil nutrition surveillance technology (p < 0.01). Lastly, a high livestock density implied a reduction in WTP for an *in-situ* soil nutrition surveillance technology (p < 0.01).

### 3.6 Perceptions toward democratization of soil nutrition data

Table 10 shows that almost all (99.3 %) farmers did not have their soil nutrition data shared on a public database. The few (0.7 %) farmers who shared their data did it within their farmer groups. The research enquired from farmers about the person they thought was suitable to access their soil nutrition data. The main persons included agricultural extension officers (26.7 %), farm owners (26 %), everyone (21 %), and fellow farmers (13.5 %). The majority (88.1 %) of the farmers perceived the democratization of data as beneficial; for instance, they cited that if an intervention agency or person had access to their soil nutrition data, they would get the relevant advice or help. In contrast, only 3.3 % thought that data sharing would raise issues such as infringement of private data.

**Table 10:** Gendered perceptions toward democratization of soil nutrition data.

Variable	Pooled	Men	Women	Youth	χ^2^
	Freq (%)	Freq (%)	Freq (%)	Freq (%)	
Soil data publicly shared	4 (0.73)	3 (0.88)	1 (0.90)	0.00	0.837
*Person to access soil nutrition data*					
Agricultural extension officer	146 (26.69)	85 (24.85)	31 (27.93)	30 (31.91)	15.669
Farm owner	142 (25.96)	95 (27.78)	31 (27.93)	16 (17.02)	
Everyone	115 (21.02)	67 (19.59)	26 (23.42)	22 (23.40)	
Fellow farmers	74 (13.53)	44 (12.87)	15 (13.51)	15 (15.96)	
No one	34 (6.22)	23 (6.73)	4 (3.60)	7 (7.45)	
Academics, scientists, universities	14 (2.56)	12 (3.51)	1 (0.90)	1 (1.06)	
County government	9 (1.65)	6 (1.75)	1 (0.90)	2 (2.13)	
Family members	9 (1.65)	6 (1.75)	2 (1.80)	1 (1.06)	
Others	4 (0.72)	4 (1.18)	0.00	0.00	
*Benefits/issues*					
Perceived soil data sharing benefits	482 (88.12)	303 (88.60)	97 (87.39)	82 (87.23)	0.202
Perceived soil data sharing issues	18 (3.29)	12 (3.51)	4 (3.60)	2 (2.13)	0.485
N	547	342	111	94	

Values in parenthesis are percentages; N represents the sample size; the P-values were computed using Pearson's χ^2^ test from a crosstabulation analysis.

## 4 Discussion

### 4.1 The existing soil nutrition management and willingness to pay for the cafetière system

The existing soil nutrition management practices revealed that farmers attempted to combat soil infertility and low pH. There was use of both inorganic and farmyard manure (FYM) among many of the households that attempted to improve the nutrition status of their soil. As much as some farmers used a combination of FYM with wood ash to control low soil pH levels, a slight majority only used FYM to control it. However, the practice of applying manure only has been reported to possibly contribute to low pH in acidic soils [54].

Low soil testing status among farmers confirms the findings of Middendorf et al. [11] and Dimkpa et al. [15]. Some of the reasons behind low soil testing, as given by farmers, are low awareness, lack of testing centers close to farmers, and high cost of soil testing [15,17]. This confirms that farmers need an alternative method to help them test their soil. We presented farmers with a power-free cafetière-style filter system to elicit their perceptions about the technology. The fact that farmers can use the method to test their soil on their farms using an affordable technology elicited a high liking for the prototype.

Willingness to pay goes beyond the 20 % premium over the cost of existing rapid tests. CVM revealed that despite the new technology bearing a premium price, they are willing to incur up to 94 % extra cost to have *in-situ* soil testing done by themselves. Young adults revealed they are ready to adopt the new technology compared to women and men farmers. In previous studies, young adults have been found to have a high likelihood of accepting new technologies [21,22,27,28]. Men face high dependency from other family members on matters other than farming enterprises. Our study shows that the households that men headed had large sizes, hence high dependence. On the other hand, women had a higher WTP than men since they are mostly fully engaged in household farming matters and consider environmental conservation more personal than men [26].

### 4.2 Effects of farm and farmer characteristics on willingness to pay for a cafetière-style filter system

The farmer's sex significantly influenced the WTP for the cafetière-style filter system prototype. Men farmers in SSA are known to have more resource endowments than their women counterparts [55]. Our findings corroborate those of Shee et al. [27] who found that the WTP of women farmers is mostly compromised by their societal status in which they are less likely to own enhancing resources such as financial capital.

Literate farmers can handle smartphones by reading instructions; thus, as farmers get to a higher educational category, they are likely to appreciate technological innovations. The findings on the positive influence of education level on WTP for agricultural technologies were also reported by others [24,26,27]. The authors acknowledge that literate farmers with primary and post-primary education are mostly the first to receive information about new technologies and adopt them first.

The majority of farmers in SSA do agriculture for subsistence [56]. This study, however, shows that when farmers conducted farming activities as business or employment, their WTP for the simple portable system increased. The findings support those of Mottaleb [23] who found that a farmer who assumes agriculture as self-employment and the main occupation has a positive significant WTP for new agricultural technologies. Kahwai et al. [28], however, found that a farmer involved in other employment off-farm has a higher WTP for new agricultural technology as it will help them manage both on-farm and off-farm activities efficiently.

Studies such as Yussif et al. [22] and Ahiale et al. [26] also found a negative influence of increasing income on WTP and adopting new technology. The explanation was that the trend might be driven by the push to invest in lucrative non-farm businesses such as real estate since the markets for agricultural products vary so often. The findings contradict those of Kahwai et al. [28], who alluded that high-income farmers have an increased capacity to purchase new technology and become early adopters.

Omotayo et al. [21] found that as the household grows bigger, it is highly likely to adopt conventional agricultural practices. This might be caused by the imbalance between expenditure on consumable goods (e.g., food) and investment in new agricultural technologies. Yussif et al. [22] reported similar findings.

Similar findings to ours, *i.e.*, that aging reduces technology adoption have been reported in the literature [21,22,27,28]. Kahwai et al. [28] reported that as farmers advance in age, they become more conservative regarding the acceptance of new technology. The research added that youthful farmers usually exhibit the swift acceptance of new agricultural technologies. Therefore, youthful farmers are likely to invest a large share of their investments in new agricultural technologies [22].

According to Ulimwengu and Sanyal [20], income diversification is expected to increase farmers’ ability to acquire new technology. However, our findings corroborate those of Yussif et al. [22] who found a negative influence of off-farm income on WTP for new technology. As such, a farmer engaging in off-farm income-earning activities is likely to make more monetary investment off-farm than on-farm. This is an area that the extension agents should capitalize on to ensure that aging farmers are not left out in the uptake of the proposed soil testing solution.

Similar to our study, households located far from the market were also previously reported by Kahwai et al. [28] to have a lower WTP for new technologies than those close to the market. The study alluded that households far from the nearest market will likely suffer from non-exposure to information on agricultural technological advancements. Farmers in such areas consider investment in new technology as an extra cost besides the costs associated with access to the far-located markets.

Our study found that farmers whose inclination is livestock production have lower WTP for the *in-situ* soil nutrition surveillance technology. The livestock production enterprise sustains soil fertility by applying FYM on fodder farms [57]. Farms, therefore, remain fertile throughout the year.

### 4.3 Democratization of soil nutrition data

Most farmers perceived the democratization of soil nutrition data as beneficial. They felt that the extension officers should have access to the general soil situation in a geographical area once farmers test their soil and the mobile app stores the results in a cloud storage. This can help the extension officers efficiently give farmers advisory services based on their understanding of soil nutrition heterogeneity [58]. Democratization would also enable soil nutrition data to be publicly available to interested stakeholders, including government and research organizations that can support farmers to improve their soil fertility status. Democratized data can make it efficient for soil scientists to compute spatial distributions of soil nutrients [17].

### 4.4 Limitations of the study

We propose a technology that would offer a possible soil testing solution to farmers in limited-resource countries who may also suffer from low literacy levels. This calls for organized pieces of training to equip farmers with the usage of the technology thus signifying extra cost implications. There is a likelihood that some farmers may capture poor-quality pictures of μPAD with their mobile phones, which may jeopardize the test results and thus get wrong recommendations. Farmers must be trained and given written instructions on the *dos and don'ts* of using the technology to get accurate results from the rapid test. The proposed soil testing innovation currently diagnoses soil macronutrients and pH leaving room for further improvement in the diagnosis of micronutrients in the future. Besides the extension officials, μPADs can only be distributed by literate persons who would help farmers understand the technology.

## 5 Conclusions and policy implications

Currently, soil testing among smallholder farmers is extremely low (1.46 %) and farmers apply fertilizers to untested soils. Some of the main reasons behind the observed low testing capacity include the lack of knowledge about soil testing, testing centers being far away from farmers, and the high costs of soil testing. We found that farmers in central Kenya are willing to pay KSh1,942.37 ($12.95) for *in-situ* measurement systems. This could address the challenges around the current low rates of farmers testing their soils. The WTP values among the youth farmers (KSh1,612.53 ($10.75)) and women (KSh1,558.68 ($10.39)) were higher than those of men (KSh1,504.83 ($10.03)). Women and young adults have lower access to assets, TLU, and land than men. Some farmers are also willing to share their soil fertility data through democratization in cloud storage. Farmers receive less than one extension visit in a year, implying that their need to have the agricultural extension officers access their soil nutrition data may be futile if the financial facilitation of extension services is not improved. Seemingly, farmers have confidence in extension services, but the officers are not easily available.

Men are more endowed with monetary resources than women; thus, they can afford the proposed technology by paying a premium price, which places a monetary value on the convenience acquired in *in-situ* soil nutrition analysis. However, they are faced with much dependency on catering to the needs of the rest of the family especially in large households. Young adults and women show high WTP for the proposed technology despite suffering from low resource endowment. As such, policy environment and development partners should focus financial resources and training more on young farmers and women to enhance their access to essential factors that might help actualize their WTP.

Literate farmers are more willing to accept and pay for technology because they are able to read and understand instructions associated with technology. It is easier for farmers who take their farming activities as agribusiness to accept the premium associated with a portable soil testing technology than their counterparts who farm for subsistence. There is a possibility that a farmer with a high income might prioritize to invest off-farm before investing in a new on-farm technology. The larger the household, the more dependency on the household head and the lower the willingness to pay for a new technology. Farmers in remote areas may suffer from low access to information on technology updates, which may lower their acceptability for the technology. On the other hand, farmers with more inclination toward livestock production may not be so willing to pay for a new soil testing technology as they do not have soil infertility problems.

The proposed prototype for *in-situ* soil nutrition surveillance technology can address the issues of portability, cost, and ease of use by farmers. Since many farmers do not know soil nutrients and pH that affect fertility, policies should be put in place to increase financial and transport facilitation for the extension officers to reach as many farmers as possible. This can increase farmers’ urge to do soil testing and mitigate soil fertility and pH accordingly, eventually increasing agricultural productivity and income.

### Ethics statement

Ethical approval for the study was granted through the Mount Kenya University Ethical Committee, ref MKU/ERC /1797 on April 30, 2021.

The informed consent was programmed into computer-assisted personal interview (CAPI) and entailed the following:

Hello! My name is ${enumname}, and we are currently conducting a survey with the goal of getting farmers' feedback regarding a potential point-of-need simple analytical tools for *in-situ* surveillance of soil nutrition in resource-limited settings. You have been randomly selected to take part in this survey, and your **VOLUNTARY** participation in this survey will be very helpful as we develop the rapid soil nutrition diagnostics tools further. Your opinion will be treated with absolute **CONFIDENTIALITY**, and the analysis of your feedback will be in combination with those of others. The findings of this survey will contribute to perfecting the development of simple tools that might be a future solution to farmers’ soil testing problems. We will just take about 30 min of your time. Are you willing to participate in this survey?

## Funding statement

This work was supported by the UK Government's Global Challenges Research Fund (GCRF) for a project titled Trialling Simple Analytical Tools for *In-situ* Surveillance of Soil Nutrition in Resource-limited Settings.

## Data availability statement

Data will be made available on request.

## Additional information

No additional information is available for this paper.

## CRediT authorship contribution statement

**Philip Kamau:** Writing – review & editing, Writing – original draft, Methodology, Formal analysis, Data curation, Conceptualization. **Ibrahim Ndirangu:** Writing – review & editing, Investigation, Formal analysis, Data curation, Conceptualization. **Samantha Richardson:** Writing – review & editing, Funding acquisition, Formal analysis, Conceptualization. **Nicole Pamme:** Writing – review & editing, Supervision, Project administration, Methodology, Funding acquisition, Conceptualization. **Jesse Gitaka:** Writing – review & editing, Supervision, Methodology, Investigation, Conceptualization.

## Declaration of competing interest

The authors declare that they have no known competing financial interests or personal relationships that could have appeared to influence the work reported in this paper.
