# Supplementary material for: Gendered farmer perceptions towards soil nutrition and willingness to pay for a cafetière-style filter system for in-situ soil testing: Evidence from Central Kenya
Source: Heliyon. 2024 Sep 11;10(18):e37568. doi: 10.1016/j.heliyon.2024.e37568 (PMC11422606; doi:10.1016/j.heliyon.2024.e37568)
Supplement: Multimedia component 1 [file mmc1.docx]

Supplementary Materials

Gendered farmer perceptions towards
soil nutrition and willingness to pay
for a cafetière-style filter system for *in-situ* soil testing*:*
evidence from Central Kenya

Philip Kamau,^a^ Ibrahim Ndirangu,^a^ Samantha Richardson,^b^ Nicole Pamme,^c*^ Jesse Gitaka^a*^

^a^ Directorate of Research and Development, Mount Kenya University,
P.O. Box 342-01000, Thika, Kenya

^b^ School of Natural Sciences, University of Hull, Cottingham Road, Hull HU6 7RX, UK

^c^ Department of Materials and Environmental Chemistry,
Stockholm Univeristy, SE-106 91 Stockholm, Sweden.

* Corresponding authors
*Email address:* [nicole.pamme@mmk.su.se](mailto:nicole.pamme@mmk.su.se) (N. Pamme), [jgitaka@mku.ac.ke](mailto:jgitaka@mku.ac.ke) (J. Gitaka)

## **ESI 1. Questionnaire**

**MOUNT KENYA UNIVERSITY AND UNIVERSITY OF HULL**

**POINT OF NEED SIMPLE ANALYTICAL TOOLS FOR IN-SITU SURVEILLANCE OF SOIL NUTRITION IN RESOURCE-LIMITED SETTINGS SURVEY 2021**

**Introduction**

We are currently conducting a survey with the goal of getting farmers’ feedback regarding a potential ***point of need, simple analytical tools for in-situ surveillance of soil nutrition in resource-limited settings***. You have been randomly selected to take part in this survey, and your **VOLUNTARY** participation in this survey will be very helpful as we develop the rapid soil nutrition diagnostics tools further. Your opinion will be treated with absolute **CONFIDENTIALITY**, and the analysis of your feedback will be in combination with those of others.

**GENERAL INFORMATION**

**Questionnaire number (QNUM) ___________________________________________**

**Enumerator ____________________________________________ ECODE_________**

**Respondent’s name (optional)______________________________________________**

**County _________________________________________________________________**

**Sub county ______________________________________________________________**

**Ward __________________________________________________________________**

**Village _________________________________________________________________**

**Category of the respondent**

**1=Men [__] 2=Women [__] 3=Youth [__]**

**Name of the household ___________________________________________________**

**Do you usually make efforts to mitigate and address the soil situation? mitgeffort__**

**1=Yes [__]**

**0=No [__]**

SECTION A: FARMER’S AWARENESS / KNOWLEDGE OF SOIL MALNUTRITION AND ACIDITY

**A1**. Do you know the **soil nutrients and pH?** **knownuts**____

1=Yes [__]

0=No [__]

**If no, proceed to A3**

**A2.** If **yes** in **A1**, please tick the items as you know them?

1=Nitrogen (N) [__]

2=Phosphorous (P) [__]

3=Potassium (K) [__]

4=Magnesium (Mg) [__]

5=Calcium (Ca) [__]

6=Sulphur (S) [__]

7=Other (specify) [__]

**A3.** Are you aware of soil **nutrients loss?**

1=Yes [__]

0=No [__]  **awareloss___**

**A4.** If **yes** in **A3**, what are the possible causes of nutrients loss? **Losscaus­­­­­­­___**

1=Soil erosion [__]

2=Underuse of fertilizers and manure [__]

3=Leaching [__]

4=High soil acidity [__]

5=Burning of the crop remains [__]

6=Continuous cropping [__]

7=Monocropping [__]

8=Other (specify) ­­­­­­­­­­­­­­­­­­­___________________

**A5.** On a scale of 1-5, how would you describe the soil nutrient levels on your farm? **nutlevs___**

1=Very poor [__] 2=Poor [__] 3 =Fair [__] 4=Good [__] 5=Very good [__]

**A6.** How did you know **about soil nutrients, pH, and nutrients loss**? **knowlsourc_______**

1=Radio [__]

2=Newspaper [__]

3=School [__]

4=Agricultural extension service [__]

5=Seminar [__]

6= Fellow farmers

7=Other (specify) ____________

****************************************************************************

PART I: QUESTIONS ON HOW FARMERS MITIGATE SOIL NUTRIENTS LOSS AND PH

SECTION B: AVAILABLE SOIL PROBLEMS REMEDY EFFORTS

**B1.** Which method (s) do you use to mitigate and address soil nutrients loss and pH? **mitmthd___**

1=Inorganic fertilizers [__]

2=Manure [__]

3=Crop residues [__]

4=Fallowing [__]

5=Compost [__]

6=Other (specify) _____ [__]

**B2.** What are the **reasons** why you **mitigate soil nutrients loss** using the methods in **B1**?

1=Health concerns [__]

2=Affordable [__]

3=Advice by an extension officer [__]

4=Low financial capabilities [__]

5=The only available in the area [__]

6=Other (specify) __________ [__]

**B3**. How **often do you usually at least mitigate nutrients loss**? **mitgoft______**

1=Monthly [__]

2=Bi-monthly [__]

3=Quarterly [__]

4=Semi-annually [__]

5=Annually [__]

6=Bi-annually [__]

7=Other (specify)___ [__]

**B4.** If the answer in **B1** is inorganic fertilizer, who mainly does the **actual purchase of the fertilizer** in your household? **fertpurch______**

1=Household head [__]

2=Spouse [__]

3=Sons/daughters [__]

4=House help [__]

5=Any HH member [__]

**B5.** Please indicate the following **details on the inorganic fertilizers** that you have purchased in the **last two seasons (2020/2021)**

| Fertilizer brand  **(Code A)** | Quantity purchased | Quantity units  1=Kgs,  2=Litres | Price per unit **(KSH)** | Outlet where **MAINLY** bought  (***one main outlet per fertilizer***)  1=NCPB  2=Agrovet  3=Open market  4=Cooperative  5=Small traders  6=Farmer groups  7=Fertilizer company agents  8=Neighbors  9=Other(specify)___________ | **Reason for choice of this outlet**  1=Reliable  2=Near to home 3=Quality products  4=Only outlet  5= Other(specify)  ________ | Level of trust on this outlet  0= None  1=Low  2=Moderate  3=High | Distance from home to the outlet **(KMS)** |
| --- | --- | --- | --- | --- | --- | --- | --- |
| **Fert** | **qtypurc** | **Units** | **price** | **Outlet** | **Choice** | **Trust** | **dist** |
|  |  |  |  |  |  |  |  |
|  |  |  |  |  |  |  |  |
|  |  |  |  |  |  |  |  |
|  |  |  |  |  |  |  |  |
|  |  |  |  |  |  |  |  |
| **Code A**: 1=DAP 7=SA (21:0:0) 13=Kero green 19= Other (specify) _______  2=MAP 8= DSP 14=Mijingu 1100  3=CAN 9= TSP 15=Mavuno basal  4=NPK 10= SSP 16=Mavuno basal  5=ASN 11=Foliar feeds 17=I can’t remember  6=UREA 12= Magmax Lime 18=I don’t know | | | | | | | |

***(ENUME: for NPK fertilizers, consider the evidence of composition of each element in the fertilizer)***

**B6**. Does any of the above-mentioned fertilizer outlets in **B5** sell using **promotional strategies** when marketing fertilizers to farmers? **prostagy**______

1=Yes [__]

0=No [__]

**B7**. If yes in **B6**, which are these **strategies?**

1 = Advertisement [__]

2 = After sales service [__]

3 = Quality display [__]

4 = Special packaging [__]

5 = Special offers [__]

6 = Other (specify) ______ [__]

****************************************************************************

PART II: SOIL NUTRITION AND PH DIAGNOSTIC TESTS

**C1.** Before using any soil nutrient replenishing method, do you usually have **your soil tested for nutrient deficiency and pH**? **soiltest______**

1=Yes [__]

0=No [__]

**If No, go to C5**

**C2.** (For yes in **C1**) Please indicate the following **details on the soil diagnostics** that you have acquired in the **last 5 years**

| Soil testing **organization or company**  1=KALRO  2=University of Nairobi  3=Crop Nutrition Laboratory Services Ltd (CROPNUTS)  4= Lagran Group Limited  5= Oxfarm Organic Ltd  6=JKUAT  7=Other (specify) __________ | Cost of soil nutrition & pH testing (KSh) | Distance from home  (Km) | Cost of public transport (KSh) | Total cost (KSh) | **Reason for choice of this outlet**  1= Reliable  2=Near to home  3= Quality products  4= Only outlet I know  5= Other (specify)  _________________ | Level of **trust** on soil tests from this premise  0= None  1=Low  2=Moderate  3=High |
| --- | --- | --- | --- | --- | --- | --- |
|  |  |  |  |  |  |  |
|  |  |  |  |  |  |  |
|  |  |  |  |  |  |  |
|  |  |  |  |  |  |  |

**C3**. As a farmer what **challenge** do you face from the soil tests by the organization or the company that performs them? **Testchal­­______**

0=None [__]

1=Expensive [__]

2=Takes time before the results are back [__]

3=Inadequate soil sampling skills [__]

4=Irregular testing [__]

5=Inconveniences cropping activities [__]

6=Other (specify) ________

**C4.** Are you **aware of any rapid soil testing** method from any company? **awaretest__**

1=Yes [__]

0=No [__]

**C5.** (For no in **C1**) If you **know about soil testing** **but do not** **access it,** what are **the reasons**?

1=Expensive [__]

2=My soil is highly fertile [__]

3=I have enough experience to diagnose the deficient nutrients [__]

4=I don’t know anything about soil testing [__]

5=The testing org/Co is far from here [__]

6= Other (specify)_______________ [__]

**C6.** (For no in **C1**) What **remedies** do you think should be **put in place for you to start the soil tests**? **remd**_____

1. ______________________________________________________________________

2. ______________________________________________________________________

3. ______________________________________________________________________

4. ______________________________________________________________________

5. ______________________________________________________________________

****************************************************************************

PART III: PERCEPTIONS AND ATTITUDES OF SIMPLE SOIL MONITORING TOOL

***(ENUMERATOR: you should carefully explain the main features of power-free cafetiere-style filter system combined with a colour readout PAD and smartphone readout to the farmers, and then ask the questions that follow)***

**The power-free cafetière system** entails simple analytical tools for rapid on-site surveillance of soil nutrition *in-situ* in resource-limited settings. It entails the use of cafetière for soil filtration and a **microfluidic paper-based analytical device (PAD**) for colorimetric readout and detection. A mobile app that interprets the findings of the PAD will help in collecting feasibility data for a future inventory that will help the government and farmers beyond this area. The system will help to detect soil nutrients (N & P) deficiency and pH. The Cafetière costs around KSh1,000 while the PADs will cost you **at most KSh100**. You will only be required to procure the cafetière once which makes soil testing easier as you will only be procuring the PADs when you need to do the diagnostic tests. The other available rapid method will require you to pay at least KSh1,000 every time you want to have some soil tests. It has to be done by a soil scientist but the system that we are proposing for you will have you do the tests on your own. The following are the **key features of the ideal soil rapid diagnostic system**:

- Cafetière
- A microfluidic PAD
- A mobile app

**D1.** We would like to capture your perceptions and attitudes about the prototype based on description above.

| **Question Number** | **Answer Options** |
| --- | --- |
| D1.1. After listening to the above description, do you think you would use this method of soil nutrient analysis? (please explain) | 1=Yes  0=No  Plus, an Open Answer if any |
| D1.2. Is this an affordable method to analyse your soil nutrients? | 1=Yes  0=No  Plus, an Open Answer if any |
| D1.3. What are the potential barriers to using this method? | 0=None  1=Initial cost  2=Sounds complex  3=I have no formal education  4=Other (specify) |
| D1.4. Would you be interested in trialling out this method? | 1=Yes  0= No |
| D1.5. If yes to **D1.4**, we may be in touch in the future regarding this method using your phone numbers. |  |
| D1.6. If no **D1.4**, why are you not interested in trialling this method? | Open Answer |

**D2**. After the description above **would you be willing** to purchase the **power-free cafetière system**? **willpurc**________

1=Yes [__]

0=No [__]

**If yes to D2, proceed to Question D4**

**D3**. If **No** in **D2** above, **give reasons** why you are **not willing to purchase** the power-free cafetière system

………………………………………………………………………………………………………………………………………………………………………………………………………………………………………………………………………………………………………………………………………………………………………………………………………………………………………………………………………………………………

**D4**. **First offer:** Considering that the available rapid test will cost you at least KSh1,000 for every soil test, will you **be willing to pay** a price of **KSh1,100** for a cafetière system that is payable once?^^[[1]](#footnote-1)^^ **offer1_____**

1=Yes [__]

0=No [__]

**D5. Second offer I:** If **Yes** in **D4** above, will you be willing to pay a price of **KSh1,200** for a cafetière system that is payable once?^^[[2]](#footnote-2)^^ **offer1_____**

1=Yes [__]

0=No [__]

**D6. Second offer II:** If **No** in **D4** above, will you be willing to pay a price of **KSh1,050** for a cafetière system that is payable once?^^[[3]](#footnote-3)^^ **offer1_____**

1=Yes [__]

0=No [__]

****************************************************************************

PART IV: PERCEPTIONS AND ATTITUDES TOWARDS THE DEMOCARTISATION OF DATA AND PROPOSED APP

***Enume: description of app***: We are thinking of developing a mobile application that compiles the data of all farm soil nutrients and pH in Kenya and sub-Saharan Africa. The app will load the coordinates of the Global Positioning System (GPS) to allow more data into a soil nutrition democratized database (cloud storage). It will be able to capture and store geotagged photos of the µPADs for analysis of the colorimetric results. This will enhance future information that is essential for tracking, navigation, and preparation of maps on soil pH and nutrition-diagnosed areas for many years. The GPS data that accumulates in the cloud database will be an avenue to inform future localized government interventions. Mapping the soil pH and nutrition for several years can help the researchers to paint a picture of the possible crop yields due to reliable information regarding soil fertility.

**SECTION E:** Please answer the following questions regarding your data

| **Question** | **Answer Options** |
| --- | --- |
| E1. Is your soil nutrient data currently shared with any other agencies? (please explain who, if your soil data is shared) | 1=Yes  2=No  Plus, open answer if any |
| E2. Is the information you share made publicly available? | 2= Don't know  1=Yes  0=No |
| E3. Who do you think should have access to soil nutrient data? (Tick all that apply) | 0=No one  1=All data should remain private to the individual farmer  2=Farm owner  3= Local farmers union groups  4= County government  5= National Government/Agricultural extension office  6= Academics, Scientists and Universities  7= Anyone / Other (please explain) |
| E4. Do you see any benefits of soil nutrient data being shared? (Please explain) | 1=Yes  2=No  plus, open answer if any |
| E5. Do you see any issues with soil nutrient data being shared? (Please explain) | 1=Yes  2=No  plus, open answer if any |

PART V: HOUSEHOLD INFORMATION

**SECTION F: CROP PRODUCTION 2020/2021**

**F1.** Do you do irrigation in your crop production?

1=Yes

2=No

**F2.** Please indicate the following **details on crop production** for the past year 2020/21

| Crop codes | Area  (a) | Land preparation  1=Manual  2=Oxen  3=Tractor | Seed type  1=Local recycled  2=Hybrid | Seeds (kg) | Harvest (kg) | Sold quantity (kg) | Sale price  (KSh/kg) | Intercropped  0=No  1=Yes | Land tenure  0=Leased  1=Own title |
| --- | --- | --- | --- | --- | --- | --- | --- | --- | --- |
| **crop** | **croparea** | **lnprep** | **sdtyp** | **sdamt** | **hvt** | **hvtsd** | **slprc** | **intcrp** | **lntnr** |
|  |  |  |  |  |  |  |  |  |  |
|  |  |  |  |  |  |  |  |  |  |
|  |  |  |  |  |  |  |  |  |  |
| Crop codes  1=Maize 6=Sorghum 11=Pyrethrum  2=Beans 7=Millet 12=Sunflower  3=Irish potatoes 8=Tomatoes 14=Garden peas  4=Sukuma wiki 9=Carrots 15=Pigeon peas  5=Cabbages 10=Wheat 16=Other (specify) | | | | | | | | | |

**SECTION G: ASSETS OWNED**

**G1.** Please give the following information regarding assets owned

| Asset  code | Quantity | Value |
| --- | --- | --- |
|  |  |  |
|  |  |  |
|  |  |  |
|  |  |  |
| **Asset code**  1=Housing (e.g., residentials, store, animal units etc…)  2=Farm equipment (e.g., jembe, panga, slasher, wheelbarrow etc. …)  3=Transportation mode (Motorcycle, car, donkey cart, bicycle etc.….)  4=Electronics (TVs, radios, fridge, mobile phones, solar units etc. …)  5=Kitchen equipment (cutlery, pestle and mortar etc.)  6=Powered machinery (tractor, power saw, generator, planter, sheller etc.)  7=Water reservoirs (dams, tanks, wells etc. …)  8=Animals (donkeys, cows, sheep etc. …)  9=Other (specify) | | |

**G2.** Farm animals owned

| Animal  code | Units | Value |
| --- | --- | --- |
|  |  |  |
|  |  |  |
|  |  |  |
|  |  |  |
|  |  |  |
| **Animal codes**; 1=Cow 6=Goose  2=Sheep 7=Duck  3=Goat 8=Rabbit  4=Chicken  5=Camel | | |

**SECTION H: DEMOGAPHIC CHARACTERISTICS (ALL FARMERS)**

**H1.** Respondent’s **gender gender**__________

1=Male [__]

0=Female [__]

**H2.** What is the **highest level of education attained**? **Educlev**________

0=No formal education [__]

1=Primary [__]

2=Secondary [__]

3=College [__]

4=University [__]

**H3.** What is your **employment status**? **Employ**________

1=Unemployed (student) [__]

2=Unemployed (Non student) [__]

3=Informal employment [__]

4=Formal employment [__]

5=Business person/ self-employed [__]

**H4.** What is the **range** of your **income per month**?  **Income**________

1=None (student) [__]

2=None (Non student) [__]

3=Low (KSh1-15,000) [__]

4=Middle (KSh15,000-50,000) [__]

5=High (> KSh50,000) [__]

**H5.** What is your **religion**? **Religion_________**

1=Catholic [__]

2=Protestant (mainstream) [__]

3=Protestant (Pentecostal) [__]

4=SDA [__]

5=Muslim [__]

6=Other (specify) ____________

**H6.** How many members are in your household? **Hhmems_________**

**H7.** Please indicate how many household members are in these age categories

**< 18 years ___________**

**18-55 years ___________**

**>55 years ___________**

**H8.** Do you earn off-farm income? **Offrminc___**

1=Yes [__]

0=No [__]

**H9.** If yes in H8, state the amount (KSh) **offarmincamt_________**

***THANK YOU FOR YOUR CO-OPERATION***

## **ESI 2. Contingent valuation model (CVM) for willingness to pay for a cafetière-style filter system for *in-situ* soil nutrition surveillance**

Regarding the cost of testing a soil sample using the only available rapid soil testing method in the study area, the first bid was KSh1,100 ($7.33), which bore a 10% premium price in comparison to a minimum of KSh1,000 ($6.67) that farmers pay for the available rapid soil testing method. If a farmer responds “yes” to the first price bid$B_{i}$, a second higher bid (KSh1,200/ $8) bearing a 20% premium price was presented$B_{i}^{u}$, where ($B_{i}^{u}>B_{i}$ ). If the consumer responds “no” to the first bid$B_{i}$, a second bid $B_{i}^{d}$ (5% premium price) was presented where ($B_{i}^{d}<B_{i}$). Therefore, with an assumption of ${WTP}_{i}\left( Z_{i},u_{i} \right)=Z_{i}^{'}\beta+u_{i}$ and $u_{i}\boldsymbol{\sim}N\left( 0,\sigma^{2} \right),$ the four outcomes expected are;

1. “yes” to first bid and second bid ($\pi^{yy}$),

$\pi^{yy}$ ($B_{i}, B_{i}^{u}$) = *Pr* (${WTP}_{i}>B_{i},{WTP}_{i}\geq B_{i}^{u}$)

= *Pr* ($Z_{i}^{'}\beta+u_{i}>B_{i},Z_{i}^{'}\beta+u_{i}\geq B_{i}^{u}$)

where${WTP}_{i}$ is the true unobserved WTP for respondent *i*, $B_{i}^{u}$ and $B_{i}^{d}$ are the price changes randomly assigned to respondent *i*, $\beta$is a vector of coefficients to be estimated for explanatory variables in vector $X$**.** Applying the Bayes rule that states *Pr* ($A,B)=\Pr(A/B)*Pr(B$), we develop the following equation (Lopez-Feldman, 2012);

$\pi^{yy}$ ($B_{i}, B_{i}^{u})=\Pr\left( Z_{i}^{'}\beta+u_{i}>B_{i}ǀZ_{i}^{'}\beta+u_{i}\geq B_{i}^{u} \right)*(Z_{i}^{'}\beta+u_{i}\geq B_{i}^{u})$

By description, $B_{i}^{u}>B_{i}$while $\Pr\left( Z_{i}^{'}\beta+u_{i}>B_{i}ǀZ_{i}^{'}\beta+u_{i}\geq B_{i}^{u} \right)*\left( Z_{i}^{'}\beta+u_{i}\geq B_{i}^{u} \right)=1$, which means that;

$\pi^{yy}$ ($B_{i}, B_{i}^{u})=Pr (u_{i}\geq B_{i}^{u}-Z_{i}^{'}\beta)$

$=1-\Phi\left( \frac{B_{i}^{u}-Z_{i}^{'}\beta}{\sigma} \right)$

Symmetrically, assuming normal distribution we end up with;

$\pi^{yy}$ ($B_{i}, B_{i}^{u})=\Phi\left( Z_{i}^{'}\frac{\beta}{\sigma}- \frac{B_{i}^{u}}{\sigma} \right)$ (1)

2. “yes” followed by “no” ($\pi^{yn}$),

$\pi^{yn}$($B_{i}, B_{i}^{u})=Pr (B_{i}{\leq WTP}_{i}<B_{i}^{u})$

$=Pr (B_{i}\leq Z_{i}^{'}\beta+u_{i}<B_{i}^{u})$

$=Pr\left( \frac{B_{i-}Z_{i}^{'}\beta}{\sigma}\leq\frac{u_{i}}{\sigma}<\frac{B_{i}^{u}-Z_{i}^{'}\beta}{\sigma} \right)$

$=\Phi\left( \frac{B_{i}^{u}-Z_{i}^{'}\beta}{\sigma} \right)-\Phi\left( \frac{B_{i}-Z_{i}^{'}\beta}{\sigma} \right)$

Following the rule that $Pr \left( a\leq X<B \right)=F\left( b \right)-F(a)$ and normal distribution symmetry we derive equation 2 for the responses following a “yes” and “no”.

$\pi^{yn}$($B_{i}, B_{i}^{u})=\Phi\left( Z_{i}^{'}\frac{\beta}{\sigma}- \frac{B_{i}}{\sigma} \right)-\Phi\left( Z_{i}^{'}\frac{\beta}{\sigma}- \frac{B_{i}^{u}}{\sigma} \right)$ (2)

3. “no” followed by “yes” ($\pi^{ny}$),

$\pi^{ny}$($B_{i}, B_{i}^{d})=Pr ({B_{i}^{d}\leq WTP}_{i}<B_{i})$

$=Pr (B_{i}^{d}\leq Z_{i}^{'}\beta+u_{i}<B_{i})$

$=Pr\left( \frac{{B_{i}^{d}}_{-}Z_{i}^{'}\beta}{\sigma}\leq\frac{u_{i}}{\sigma}<\frac{B_{i}-Z_{i}^{'}\beta}{\sigma} \right)$

$=\Phi\left( \frac{B_{i}-Z_{i}^{'}\beta}{\sigma} \right)-\Phi\left( \frac{B_{i}^{d}-Z_{i}^{'}\beta}{\sigma} \right)$

Following the normal distribution symmetry;

$\pi^{ny}$($B_{i}, B_{i}^{d})=\Phi\left( Z_{i}^{'}\frac{\beta}{\sigma}- \frac{B_{i}^{d}}{\sigma} \right)-\Phi\left( Z_{i}^{'}\frac{\beta}{\sigma}- \frac{B_{i}}{\sigma} \right)$ (3)

4. “no” for both responses ($\pi^{nn}$),

$\pi^{nn}$($B_{i}, B_{i}^{d})=Pr({WTP}_{i}<B_{i},{WTP}_{i}\leq B_{i}^{d})$

$=Pr(Z_{i}^{'}\beta+u_{i}<B_{i},Z_{i}^{'}\beta+u_{i}\leq B_{i}^{d})$

$=Pr(Z_{i}^{'}\beta+u_{i}<B_{i}^{d})$

$=\Phi\left( \frac{B_{i}^{d}-Z_{i}^{'}\beta}{\sigma} \right)$

Following the normal distribution symmetry;

$\pi^{nn}$($B_{i}, B_{i}^{d})=1-\Phi\left( Z_{i}^{'}\frac{\beta}{\sigma}- \frac{B_{i}^{d}}{\sigma} \right)$ (4)

There is no preexistent model such as Probit or Logit that can estimate WTP using equations 1 to 4 unlike if we used a single bounded CVM. We used maximum likelihood estimation (MLE) to directly acquire the value of $\beta$ and $\sigma$ following the novel Lopez-Feldman likelihood function. To obtain the relevant parameters, we maximized the following function;

$$\sum_{i=1}^{N} \left[ \pi^{yy}\ln\left( \Phi\left( Z_{i}^{'}\frac{\beta}{\sigma}- \frac{B_{i}^{u}}{\sigma} \right) \right)+\pi^{yn}\ln\left( \Phi\left( Z_{i}^{'}\frac{\beta}{\sigma}- \frac{B_{i}}{\sigma} \right)-\Phi\left( Z_{i}^{'}\frac{\beta}{\sigma}- \frac{B_{i}^{u}}{\sigma} \right) \right)+\pi^{ny}\ln\left( \Phi\left( Z_{i}^{'}\frac{\beta}{\sigma}- \frac{B_{i}^{d}}{\sigma} \right)-\Phi\left( Z_{i}^{'}\frac{\beta}{\sigma}- \frac{B_{i}}{\sigma} \right) \right)+\pi^{nn}\ln\left( 1-\Phi\left( Z_{i}^{'}\frac{\beta}{\sigma}- \frac{B_{i}^{d}}{\sigma} \right) \right) \right]$$

 (5)

Where a farmer enters once in each of the relevant cases $\pi^{yy}$, $\pi^{yn}$, $\pi^{ny}$, and $\pi^{nn}$ though 1 or 0 dummy in the logarithmic likelihood function. $\Phi$ represents the standard normal cumulative distribution function. The MLE directly evaluates $\hat{\beta}$and $\hat{\sigma}$, mean WTP for a simple affordable portable soil testing tool is the constant of the likelihood function for an equation with no control variables. Estimating WTP in an equation using control variables involves creating scalar values for each explanatory variable. Supplementary Table 2 shows the variables used in the measurement of WTP in CVM, their description, and expected signs.

## **ESI 3. Variables used in the contingent valuation method model**

**Table ESI1.** Variables used in the contingent valuation method model

| Variable | Description and measurement of variable | A priori sign |
| --- | --- | --- |
| **Dependent variables** | | |
| WTP | Willingness to pay for the proposed system in Kenya Shillings (KSh):   - 1=yy if yes to first and second bid; 0=otherwise - 1=yn if yes to first bid and no to second bid; 0=otherwise - 1=ny if no to the first bid and yes to the second bid; 0=otherwise - 1=nn if no to first and second bid; 0=otherwise |  |
| **Independent variables** | | |
| Sex | Gender of the HHH is a binary variable: 1=Male; 0=Female | + |
| Education | Education of the HHH is a dummy variable: 2= Post-primary education; 1=Primary education; 0=No formal education | +/- |
| Employment | HHH employment type is a dummy: 3=Formal employment; 2=Informal employment; 1=Unemployed | + |
| Income | Monthly income of the HHH is a dummy: 3= Above KSh50000; 2=KSh15000 – 5000; 1=KSh0 – 15000 | + |
| Off-farm income | Off-farm income is a binary variable: 1=HHH received off-farm income; 0=otherwise | + |
| Household size | Household size is a continuous number | +/- |
| Group membership | Membership to an organized association/group is a binary variable: 1=HHH belonged to a group; 0=otherwise | + |
| Land tenure | Land tenure is a binary variable: 3=Owned titled and leased; 2=Owned title; 0=Leased | + |
| Credit access | Access to credit is a binary variable: 1=HHH received credit for agricultural use; 0=otherwise | + |
| Distance to market | Distance to the nearest input/product market measured in kilometers | - |
| Extension contacts | Number of times an agricultural extension officer visited the farm from April 2020 to February 2021 | + |
| TLU | Tropical livestock units measured as an index: cow (0.7); goat/sheep (0.1); chicken/fish (0.01); camel (1.1); goose/turkey/duck (0.03); rabbits (0.02); pig (0.2) | +/- |
| Gender category | Gender category which the HHH belong is a dummy variable: 3=Youth; 2=Women; 1=Men | +/- |
| Area under crops | Area under crop production measured in acres | +/- |
| Irrigation | If the household does crop production under irrigation: 1=A household does irrigation; 0=otherwise | + |

## **ESI 4. Multicollinearity diagnosis for contingent valuation method (CVM) model**

**Table ESI2**. Variance inflation factor (VIF)

| Variable | VIF | 1/VIF |
| --- | --- | --- |
| Own land titles | 9.77 | 0.102318 |
| Own land titles and lease | 9.53 | 0.104879 |
| Primary education | 6.95 | 0.143820 |
| Post-primary education | 6.86 | 0.145821 |
| TLU | 4.34 | 0.230450 |
| Credit | 3.83 | 0.260759 |
| Self-employed | 3.79 | 0.264000 |
| Age (36-55 years) | 3.34 | 0.299667 |
| Formal employment | 3.28 | 0.305083 |
| Age (18-35 years) | 3.12 | 0.320514 |
| Distance to market | 3.01 | 0.332000 |
| Extension education | 2.6 | 0.384053 |
| Irrigation | 2.39 | 0.418805 |
| Off-farm income | 1.69 | 0.590138 |
| Group membership | 1.64 | 0.611260 |
| Household size | 1.6 | 0.623101 |
| Middle income (KSh15000-50000) | 1.39 | 0.722016 |
| Acres under crop | 1.31 | 0.761464 |
| High income (> KSh50000) | 1.3 | 0.771306 |
| Women | 1.23 | 0.814616 |
| Youth | 1.14 | 0.874000 |
| Sex | 1.1 | 0.912004 |
| Mean VIF | 3.42 |  |

Note: The mean variance inflation factor (VIF) for individual variables, as well as the overall mean VIF, are all less than 10. The corresponding statistical formula is presented in the main manuscript (section 2.4.2).

## **ESI 5. Findings**

**Table ESI3.** Influence of control variables on WTP for the new soil testing technology using contingent valuation method

| Variable | Coef. | Std. Err. | z | P>\|z\| |
| --- | --- | --- | --- | --- |
| Sex | 83.09** | 38.92 | 2.13 | 0.033 |
| *Education* |  |  |  |  |
| Primary education | 151.85*** | 44.59 | 3.59 | 0.000 |
| Post primary education | 204.14*** | 56.84 | 3.59 | 0.000 |
| *Employment* |  |  |  |  |
| Self-employment | 174.33** | 74.37 | 2.34 | 0.019 |
| Formal employment | 43.21 | 80.51 | 0.54 | 0.591 |
| *Income* |  |  |  |  |
| Middle (KSh15,000-50,000) | -47.46 | 54.70 | -0.87 | 0.386 |
| High (> KSh50,000) | -256.64*** | 87.80 | -2.92 | 0.003 |
| Household size | -24.24*** | 9.12 | -2.66 | 0.008 |
| *Age* |  |  |  |  |
| 36-55 years | 233.66*** | 71.98 | 3.25 | 0.001 |
| > 55 years | 134.14*** | 47.80 | 2.81 | 0.005 |
| Off-farm income | -73.23* | 42.67 | -1.69 | 0.088 |
| Group membership | 1.55 | 43.96 | 0.04 | 0.972 |
| *Land tenure* |  |  |  |  |
| Own title | 1041.02 | 34564.52 | 0.03 | 0.976 |
| Title + lease | 869.06 | 34564.37 | 0.03 | 0.980 |
| Credit access | 48.24 | 89.54 | 0.54 | 0.590 |
| Distance to market | -28.65*** | 10.70 | -2.68 | 0.007 |
| Extension contacts | -0.76 | 11.27 | -0.307 | 0.946 |
| TLU | -219.12*** | 59.85 | -3.66 | 0.000 |
| *Gender category* |  |  |  |  |
| Women | 5.62 | 59.88 | 0.09 | 0.925 |
| Youth | 64.55 | 78.62 | 0.82 | 0.412 |
| Area under crops | 0.73 | 9.17 | 0.08 | 0.937 |
| Irrigation | 34.62 | 73.54 | 0.47 | 0.638 |
|  |  |  |  |  |
| Model summary |  |  |  |  |
| Log-likelihood | -67.16 |  |  |  |
| Wald χ^2^ (23) | 40.84 |  |  |  |
| Prob > χ^2^ | 0.008*** |  |  |  |
| No. of observations | 547 |  |  |  |
| First-Bid Variable: bid1 |  |  |  |  |
| Second-Bid Variable: bid2 |  |  |  |  |
| First-Response Dummy Variable: answer1 | | |  |  |
| Second-Response Dummy Variable: answer2 | | |  |  |

^***^, ^**^ and ^*^ are statistical significance at 1%, 5% and 10%, respectively. The coefficients were estimated using maximum likelihood estimation (MLE) after incorporating the explanatory variables in the *doubleb* module.

## **ESI 6. χ^2^ test on reasons given by the surveyed farmers for not testing their soils**

**Table ESI4.** χ^2^ test results for no soil testing

|  | Men | Women | Youth | Total |
| --- | --- | --- | --- | --- |
| Expensive | 70 | 11 | 10 | 91 |
| Good soils | 10 | 1 | 3 | 14 |
| Personal diagnosis of soil malnutrition through crop color changes | 3 | 0 | 0 | 3 |
| Lack knowledge about soil testing | 104 | 52 | 39 | 195 |
| Testing centres far away from home | 123 | 34 | 28 | 185 |
| Lack of interest | 32 | 13 | 14 | 59 |
| Total | 342 | 111 | 94 | 547 |
| Pearson chi2(10) = 22.1893 Pr = 0.014 | | |  |  |

The Pearson χ^2­^ was computed through tabulation and including the chi test in the Stata syntax.

1. 10% premium for convenience and possible sustainability of the diagnostics [↑](#footnote-ref-1)
2. 20% [↑](#footnote-ref-2)
3. 5% [↑](#footnote-ref-3)
